# Differential Response of Phenol Metabolism Associated with Antioxidative Network in Elicited Grapevine Suspension Cultured Cells under Saline Conditions

**DOI:** 10.3390/antiox11020388

**Published:** 2022-02-15

**Authors:** Lorena Almagro, Antonio A. Calderón, María A. Pedreño, María A. Ferrer

**Affiliations:** 1 Departamento de Biología Vegetal, Campus Universitario de Espinardo, Universidad de Murcia, 30100 Murcia, Spain; lorena.almagro@um.es (L.A.); mpedreno@um.es (M.A.P.); 2 Departamento de Ingeniería Agronómica, Universidad Politécnica de Cartagena, Paseo Alfonso XIII 48, 30203 Cartagena, Spain; antonio.calderon@upct.es

**Keywords:** antioxidative metabolism, elicitors, gene expression, phenolic metabolism, salt treatment, *Vitis vinifera* (L.) suspension cultured cells

## Abstract

Highly productive *trans*-resveratrol (*t*-R) grapevine suspension cultured cells (SCC) and two effective elicitors, methyl jasmonate (MJ) and randomly methylated β-cyclodextrins (CDs), were used to analyze the extent to which salt treatments alter the production of bioactive phenolic compounds. The expression/activity profile of the enzymes involved in phenol metabolism and antioxidant networks were also studied. A marked extracellular accumulation of phenolic compounds, especially *t*-R, was found in SCC elicited with CDs and/or MJ under saline conditions. However, the treatments with MJ alone and all those combined with salt favored the intracellular accumulation of catechin and ferulic acid. The salt-induced accumulation of phenolics was correlated with the higher total antioxidant capacity values found in cells, suggesting that cellular redox homeostasis under saline conditions was largely maintained by increasing phenolic compound production. These higher levels of phenolics found in elicited cells under saline conditions fit well with the highest activity of phenylalanine ammonia-lyase. Moreover, antioxidant enzyme activities were boosted in treatments with MJ and/or in those combined with salt and decreased in those treated with CDs. These results suggest a differential response of the antioxidative network to the presence of elicitors under saline conditions.

## 1. Introduction

*trans*-Resveratrol (3,4′,5-trihydroxystilbene; *t*-R) is a polyphenolic compound of a stilbene nature found in several dietary plants, such as plums, red berries, peanuts, or grapes, and some beverages, such as red wine [1,2]. In plants, *t*-R is involved in the constitutive and inducible defense responses against both biotic and abiotic stresses [3,4]. *T*-R is also well known for its relevant benefits on human health [5]. The remarkable bioactivity of *t*-R has prompted much research attention, mainly after the pioneering study of Pezzuto’s group [6], and includes the prevention of many diseases, such as cancer, diabetes, neurodegeneration, aging related disorders, and cardiovascular diseases [5]. Its health-promoting effects are well-correlated with its cell defense properties [7]. In line with this, *t*-R can directly act as a scavenger of reactive oxygen/nitrogen species, or can indirectly enhance the antioxidative pathways responsible for the maintenance of cellular redox homeostasis [7]. Due to its therapeutic potential, the demand for *t*-R keeps on rising for pharmaceutical, nutraceutical, and cosmetic uses [8]. An efficient system for the production of *t*-R consists in the use of plant cell cultures as biofactories under elicitation [9]. Our group has reported production values of *t*-R close to 4 g L^−1^ in suspension cultured cells (SCC) of *Vitis vinifera* cv. Monastrell by the simultaneous addition of the stress-related hormone methyl jasmonate (MJ) and randomly methylated β-cyclodextrins (CDs) [10]. The potential role of CDs to elicit plant defense responses was first reported by our team in 1998 [11]. Since that time, similarly successful results in the production of bioactive compounds such as phytosterols, taxanes, taraxasterol, and solavetivone, among others using CDs, have been described in other plant SCC [12,13,14,15]. These successful production levels of bioactive compounds are due to the special characteristics that CDs have. The chemical structure of CDs makes them special, not only as elicitors, but also as compounds capable of trapping highly hydrophobic molecules such as *t*-R inside them [16].

On the other hand, one of the early events triggered upon elicitation, and under almost any stress conditions, is the overproduction of reactive oxygen species (ROS) in plant cells [17,18,19]. Current evidence strongly supports that low levels of ROS function in cells as signal transduction molecules that initiate transcriptomic changes that ultimately lead to an appropriate response against specific stressors or stimuli [20]. Studies carried out on elicited cell cultures from different plant species have also shown the dependence of H_2_O_2_ production on the accumulation of phytoalexins [14,15]. Changes in a plant’s metabolome are somehow linked to ROS accumulation and ROS-dependent signaling [17,20,21,22,23]. In plant cells, ROS levels are controlled by a complex network of antioxidants and antioxidant enzymes [23]. Key antioxidant enzymes include superoxide dismutase (SOD), which converts superoxide radicals to hydrogen peroxide and oxygen, and H_2_O_2_-detoxifying enzymes. The latter include catalase (CAT), the four enzymes involved in the ascorbate-glutathione (ASC-GSH) cycle (i.e., ascorbate peroxidase (APX), monodehydroascorbate reductase (MDHAR), dehydroascorbate reductase (DHAR), and glutathione reductase (GR)), as well as the class III plant peroxidases (PRXs) [19,23]. Very recently, we have reported the involvement of H_2_O_2_ in the accumulation of *t*-R in Monastrell SCC elicited with MJ and/or CDs [24]. However, information about the effects of elicitor treatments on the expression/activity of ROS-scavenging enzymes in cell cultures is still scarce.

SCC constitute a well-suited system for studying the defense responses to a variety of stressors because antioxidants’ systems can be synchronously activated in in vitro cultured cells [25]. In grapevines, it has been described that jasmonates (JA) enhanced the formation of stilbenes upon different stress conditions, including fungal infection [26], UV irradiation [27], and salt stress [28]. Whereas the effect of fungi and UV light treatments in the transcriptional control of genes involved in *t*-R synthesis is well documented, much less is known about the effects of salt on the induction of *t*-R biosynthetic/metabolic genes. The biosynthesis of *t*-R and most phenolic compounds branches from the core phenylpropanoid pathway, in which the amino acid phenylalanine is transformed into 4-coumaroyl-CoA [29]. Then, this activated CoA ester is condensed with three malonyl-CoA units to produce either naringenin chalcone, by the action of chalcone synthase (CHS), or *t*-R, by the action of stilbene synthase (STS) [29]. Since both CHS and STS are structurally similar and compete for the same substrates, they could be differentially affected in response to stressors/elicitors in order to regulate the metabolic flux between flavonoids and stilbenes [30,31]. Emerging data indicate that phenylpropanoids play a critical role in the tolerance of plants to environmental constraints, including salinity [32,33,34]. Among their multiple roles, phenylpropanoids can both avoid ROS production and quench ROS once they are produced, thereby, they can modulate ROS-dependent signaling [32].

Since salinity involves the overproduction of ROS, which can cause cell oxidative damage [35], the activation of enzymatic and non-enzymatic antioxidant compounds is considered essential to ensure cellular redox homeostasis and to produce appropriate responses [35,36]. Apart from ROS, JA biosynthesis has been involved in the cellular adaptation to saline tolerance [28,37,38]. Therefore, the aim of the present work is to analyze the extent to which the production of bioactive phenolic compounds and the expression/activity of the key enzymes involved in both phenolic metabolism and antioxidant networks are affected under saline conditions by single and double combinations of MJ and CDs in Monastrell SCC. As salinity is one of the major abiotic stressors limiting crop productivity worldwide [39], the results derived from this work may contribute to the design of strategies to improve both the accumulation of bioactive compounds and tolerance to saline conditions, and allow for progress in understanding how the antioxidant mechanisms act.

## 2. Materials and Methods

### 2.1. Plant Material and Elicitation Treatments

Grapevine SCC (*Vitis vinifera* L. cv. Monastrell from Albacete, Spain (38°47′22″ N, 1°41′09″ W)) were routinely maintained and cultured as previously described [40,41]. For the elicitation experiments, 4 g fresh weight (FW) of cells in the stationary-phase culture (15 d) were transferred to 100 mL flasks that contained 20 mL of the culture medium, supplemented with a single, double, or triple combination of NaCl (50 mM), CDs (50 mM; Wacker Chemie, Madrid, Spain), and MJ (0.1 µM; Sigma-Aldrich, Madrid, Spain). The elicited-SCC and control treatments were maintained in dark conditions in a rotary shaker (110 rpm; Orbitron, Infors HT, Bottmingen, Switzerland) at 25 °C [41,42].

The cells were collected by filtration on days 1, 3, and 4 after elicitation. Then, the cells were immediately washed with cold distilled water, weighted and frozen in liquid nitrogen, and kept at −80 °C until further analysis. The sensitivity of Monastrell SCC to salt was previously determined by measuring cell growth in culture media supplemented with different NaCl doses (0, 25, 50, 75, and 100 mM). NaCl doses > 50 mM strongly reduced cell growth, as previously observed [28]. Cell viability was measured using two fluorescent probes, fluorescein diacetate for detecting living cells, and propidium iodide for identifying dead cells, as previously described [43].

### 2.2. Quantitative Analyses of Antioxidant Capacity, Phenolic Compounds, and Trans-Resveratrol

The liquid nitrogen-frozen cells (~200 mg) were mixed with 100% methanol (1:2, *w*/*v*), ultrasonicated (30 min at 40 °C), and centrifuged (15,000× *g* for 15 min at 4 °C). The cell-methanolic extracts and the extracellular medium were used to determine the total antioxidant capacity (TAC) and the content of total soluble phenolic compounds (TPC). The TAC was assessed using the stable, colored-radical 2,2-diphenyl-1-picrylhydrazyl radical (DPPH) and Trolox, a water-soluble equivalent of vitamin E, as standard [44]. The TAC was expressed as mol of Trolox equivalent antioxidant capacity (TEAC) per gram of cell dry weight (DW). The TPC were determined using the Folin-Ciocalteu method using gallic acid as standard [45]. The results were expressed as mol of gallic acid equivalents per gram of DW (GAE g^−1^ DW).

*t*-R was extracted from both the culture media and the cells as previously described [42]. *t*-R quantification was carried out by HPLC-DAD (Waters 600E, Waters 996, Milford, Massachusetts, USA) using a Spherisorb ODS2 C-18 column (250 mm × 4.6 mm, 5 µm) and a mobile phase consisting of 0.05% trifluoroacetic acid (TFA) (solvent A) and 0.05% TFA in methanol/acetonitrile 60:40 (*v*/*v*) (solvent B) at a flow rate of 1.0 mL min^−1^. The solvents used were 0 min, 10% B; 5 min, 15% B; 40 min, 35% B; 45 min, 65% B; 50 min, 65% B; and 55 min, 10% B [9]. *t*-R was identified at 304 nm and quantified by comparison with its commercial standard (Sigma-Aldrich, Madrid, Spain).

### 2.3. Enzyme Assays

The liquid nitrogen-frozen cells (1 g) were homogenized in 2 mL of ice-cold 100 mM potassium phosphate buffer (pH 7.8) containing 1 mM of EDTA, 5 mM of MgCl_2_, 5 mM of ascorbate, 2 mM of cysteine, 1 mM of PMSF, and 0.2% (*w*/*v*) Triton X-100. After centrifugation at 14,000× *g* for 20 min at 4 °C, a 1.5 mL of supernatant fraction of each sample was desalted on NAP-25 columns (GE Healthcare). The protein extracts were quantified using the Bradford protein assay kit (Bio-rad Laboratories, Hercules, CA, USA) using BSA as a standard.

The activity of phenylalanine ammonia-lyase (PAL, EC 4.3.1.24) was determined by the conversion of L-phenylalanine into *trans*-cinnamic acid (ε_290_ = 9.5 mM^−1^ cm^−1^) [46]. Polyphenol oxidase (PPO, EC 1.14.18.1) activity was followed at 400 nm in a reaction medium containing 0.02% (*v*/*v*) sodium dodecyl sulfate (SDS) and 3 mM of *tert*-butylcatechol in 100 mM of phosphate buffer; pH 7.0 (ε_400_ = 1682 M^−1^ cm^−1^) [47]. Class III peroxidase (PRX, EC 1.11.1.7) activity was estimated using 70 μM of *t*-R (ε_305_ = 26.8 mM^−1^ cm^−1^) and 320 μM of 3,3′,5,5′-Tetramethylbenzidine (TMB) (ε_652_ = 39 mM^−1^ cm^−1^) as electron donors [48,49]. Glutathione-*S*-transferase (GST, E.C. 2.5.1.18) was determined spectrophotometrically at 340 nm by monitoring the formation of conjugates between GSH and 1-chloro,2,4-dinitrobenzene (CDNB, Sigma-Aldrich, Madrid, Spain) in a reaction medium containing 1.0 mM of GSH and 1.0 mM of DCNB in 100 mM of phosphate buffer; pH 7.5 (ε_340_ = 9,6 mM^−1^ cm^−1^) [50]. Catalase (EC 1.11.1.6) activity and the ASC-GSH cycle enzymes (APX, EC 1.11.1.11; MDHAR, EC 1.6.5.4; DHAR, EC 1.8.5.1; and GR, EC 1.6.4.2) were determined as previously described [51]. SOD (EC 1.15.1.1) activity was measured using the phenazine methosulfate (PMS)-NADH system [52]. One unit of SOD was defined as the amount of enzyme that inhibits the reduction rate of nitroblue tetrazolium chloride (NBT) by 50% under the assay conditions.

### 2.4. Quantitative Real-Time RT-PCR (qRT-PCR)

The total RNA of cells was extracted using a Trizol reagent (Invitrogen, Madrid, Spain) following the manufacturer’s protocol. The concentration of RNA in each sample was measured with a NanoDrop^®^ ND-2000 Spectrophotometer (Thermo Scientific, Waltham, MA, USA). First-strand cDNA was synthesized using the RevertAid First Strand cDNA Synthesis Kit (Thermo Scientific, Madrid, Spain). qRT-PCR was performed with the SYBR Green PCR Core Reagents Kit (Life Technologies, Madrid, Spain) using specific primers (described in Table S1). The reaction conditions and primer amplification efficiency were performed as previously reported [53]. As an internal reference, the elongation factor alpha 1 (*EFα1*) gene was used [10]. The relative expression levels were calculated by using the 2^−ΔΔ*CT*^ method [54]. An expression analysis of each time point was repeated at least three times. To make data distribution symmetrical, the gene expression ratio values (treated/untreated control) were log2-transformed.

### 2.5. HPLC-MS Analysis of Phenolic Compounds

The individual phenolic compounds were analyzed at 72 h using a HPLC-MS system (Agilent Series 1200, Agilent Technologies, Santa Clara, CA, USA). For this, separation was carried out on a C18 column (4.6 mm × 250 mm, 5 µm) at 25 °C, as has been previously described [55]. The mobile phase consisted of solvent A (formic acid 0.5%) and solvent B (acetonitrile-formic acid 0.1%). The gradient was as follows: 0 min, 2% solvent B; 10 min, 20% solvent B; 36–37 min, 100% solvent B; 37.5 min, 2% solvent B; and 40 min, 2% solvent B. The flow rate was 0.8 mL/min, and the injection volume was 20 μL. Mass spectral analysis was performed using a TOF/Q-TOF MS (Agilent Series 6220, Agilent Technologies, Santa Clara, CA, USA) equipped with an ESI operating in negative ion mode, using the following operation parameters: the capillary spray, fragmentor, and octopole RF voltages were 2500 V, 180 V, and 250 V, respectively; the nebulizer pressure was 60 psi; the drying gas flow was 12 L/min; and the drying gas temperature was 350 °C. The MS mass range was set up at 50–1200 *m*/*z* with a scan rate of 1.9 spectra/s.

The quantification of phenolic compounds was performed by using external standards for *p*-coumaric acid (assay ≥ 98%), chlorogenic acid (assay ≥ 95%), ferulic acid (assay ≥ 99%), piceid (assay ≥ 95%), and (+)-catechin (assay ≥ 98%) (Sigma-Aldrich, Madrid, Spain). All of the standard calibration curves were made using a concentration range from 0.01 to 1 ppm, with the exception of piceid (0.01–10 ppm), and showed good linearity (r^2^ > 0.99) between the standard amount and the peak area in the chromatograms. All experiments were performed in triplicate.

### 2.6. Statistical Analysis

Data were shown as the mean values ± standard errors (SE). An analysis of variance (ANOVA) for comparing the treatment means was tested by Tukey’s honestly significant difference (HSD) test, available in the SPSS statistical package (version 26.0; SPSS Inc., Chicago, IL, USA). Differences were considered statistically significant at *p* < 0.05. Data were also evaluated by principal component analysis (PCA) using the CANOCO software (version 4.5, Microcomputer Power, Ithaca, NY, USA). Prior to PCA, the raw data were log(x + 1) transformed and median centered.

## 3. Results

### 3.1. Effects of Elicitors on Cell Growth and Viability under Salt Conditions

**Figure 1:**
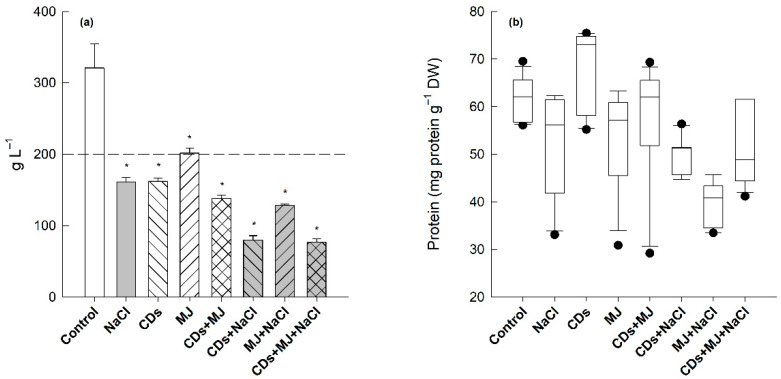
The effects of salt and/or elicitor treatments on (**a**) cell biomass accumulation (g L^−1^) and (**b**) total protein content in Monastrell suspension cultured cells (SCC) (mg protein g^−1^ FW). 15-day-old suspension cell cultures were maintained for 4 d without elicitors (control) or supplemented with a single, double, or triple combination of NaCl (50 mM), CDs (50 mM), and methyl jasmonate (MJ) (0.1 µM). Data represent the mean ± SE of three independent experiments. Treatments marked with an asterisk are significantly different from the control (*p* < 0.05) by Tukey’s honestly significant difference (HSD) test. Protein values are expressed as box-and-whisker plots. The box represents the interquartile range (IQR), the bold line in box represents the median, the whiskers represent 1.5 times the IQR, and the single dots (•) represent outlier points.

After 4 days of exposure to 50 mM of NaCl, the biomass production of grapevine SCC decreased to 50% (Figure 1a). This reduction in cell growth capacity was similar to that observed in single and double combinations of MJ and CDs. However, the presence of salt in combination with CDs or CDs + MJ led to a further decrease in cell biomass (>75%). MJ-treated cells maintained their initial cell density (200 g FW L^−1^) after 4 days of cultivation. Despite the decrease in cell biomass production, no significant changes were observed in either total protein content (Figure 1b), used as a general indicator of cell metabolism [56], or cell viability, assessed by fluorescein diacetate, between the controls and the elicitors or saline treatments (data not shown).

### 3.2. Effects of Elicitors on Cell Antioxidant Properties, Phenolics and t-R Production under Salt Conditions

**Figure 2:**
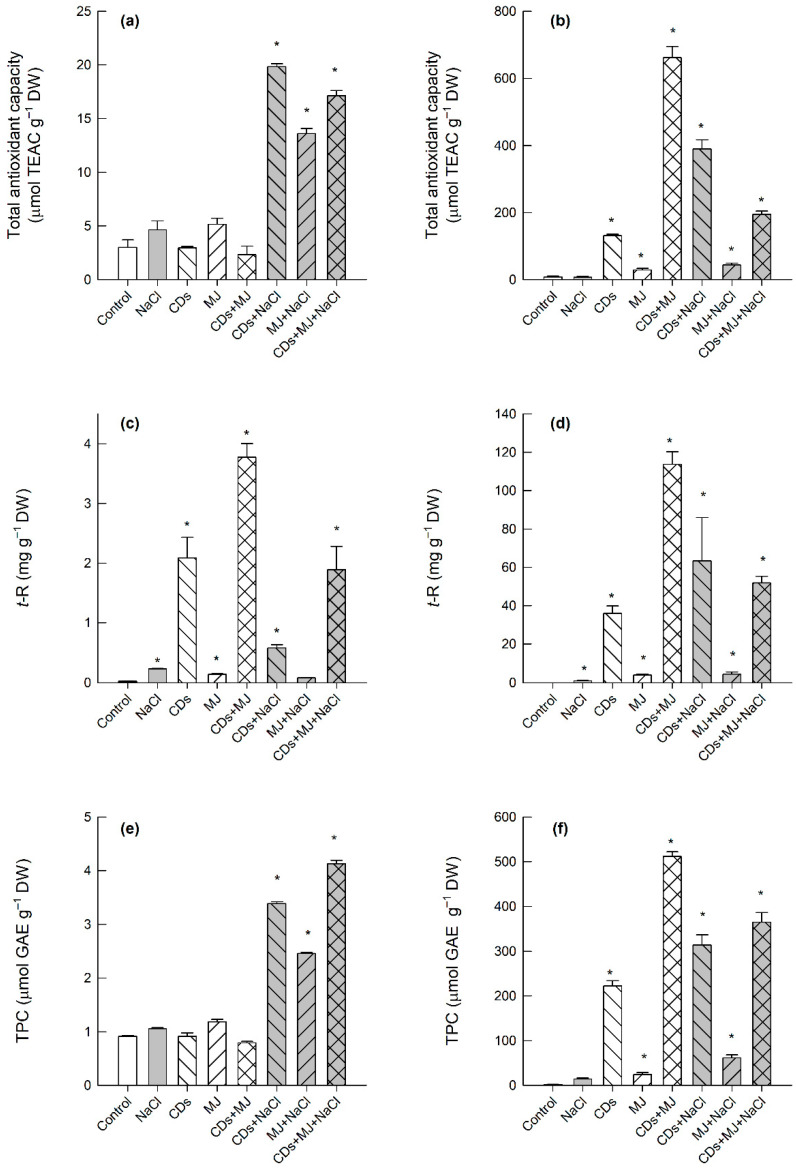
The effects of salt and/or elicitor treatments on the total antioxidant capacity (TAC) (**a**,**b**), *t*-R levels (**c**,**d**), and the total phenolic content (TPC) (**e**,**f**) in intracellular (left; (**a**,**c**,**e**)) and extracellular (right; (**b**,**d**,**f**)) extracts of Monastrell SCC. The data represent the mean ± SE of three independent experiments. Treatments marked with an asterisk are significantly different from the control (*p* < 0.05) by Tukey’s HSD test.

Since elicitor and salt treatments are known to affect ROS production and influence cell redox status [17,35], the intra- and extra-cellular antioxidant capacities were evaluated in order to have a first evaluation of the antioxidant properties of grapevine SCC. No statistically significant changes in intracellular TAC were found in the presence of single elicitor treatments or in CDs + MJ-treated cells (Figure 2a). However, a strong increase in TAC was observed in all salt-treated elicited-cultures, particularly in those combined with CDs (Figure 2a). In turn, all spent media from elicited cultures showed higher antioxidant capacity than those of controls. The maximum extracellular TAC values were found in the presence of CDs either in combination with MJ or NaCl (Figure 2b).

As phenolic compounds are one of the main antioxidant-specialized metabolites present in grapevine [57], the intra- and extracellular content of *t*-R and of TPC were analyzed. The salt treatment alone provoked not only an increase in the accumulation of *t*-R inside the cells (~230 μg g^−1^ DW), but also its accumulation in the spent media (~1 mg g^−1^ DW) (Figure 2c,d). As expected, the highest intra- and extracellular *t*-R levels were obtained in the double combination CDs + MJ treatments (~4 and ~120 mg g^−1^ DW, respectively). A high extracellular *t*-R production was observed in salt-treated cells in the presence of CDs (~63 mg g^−1^ DW) and CDs + MJ (~52 mg g^−1^ DW). The accumulation of *t*-R in the spent media followed the same pattern as the extracellular TAC values (Figure 2b,d), and a strong correlation between both parameters was found (r > 0.96, *p* < 0.01) (Table S2).

Regarding the production of soluble phenols, a marked increase in the intracellular concentration of phenolics in all of the salt-elicited treatments was noticed (Figure 2e). It is important to highlight that the pattern of intracellular TPC (Figure 2e) was similar to those of intracellular TAC (Figure 2a), whereas the extracellular TPC pattern (Figure 2f) resembled those of both extracellular *t*-R content (Figure 2d) and TAC (Figure 2b). Similarly, strong correlations between these parameters were also found (r > 0.93, *p* < 0.01) (Table S2).

Since elicitation treatments in salt-treated cells provoked a striking rise in intracellular TPC and TAC levels, HPLC-MS analyses were performed to identify and quantify the main phenolics that accumulated in response to each treatment. As depicted in Table 1, only *trans*-piceid (resveratrol 3-β-glucoside) was detected in untreated cell cultures. Salt treatment alone, however, provoked a drop of ~50% in intracellular piceid content, as well as an increase in the levels of hydroxycinnamic acids (HCAs), i.e., *p*-coumaric acid, chlorogenic acid, and ferulic acid. Chlorogenic acid, ferulic acid, and (+)-catechin were not detected in CDs-treated cells under non-saline conditions. Nonetheless, the levels of HCAs and (+)-catechin tended to increase in salt-treated cells in the presence of elicitors, either alone or in combination, whereas the levels of piceid tended to decrease, with the exception of the MJ single treatments, in which piceid levels were similar to the untreated controls (Table 1).

### 3.3. Effects of Elicitors on Phenol- and t-R-Metabolizing Enzymes under Salt Conditions

**Figure 3:**
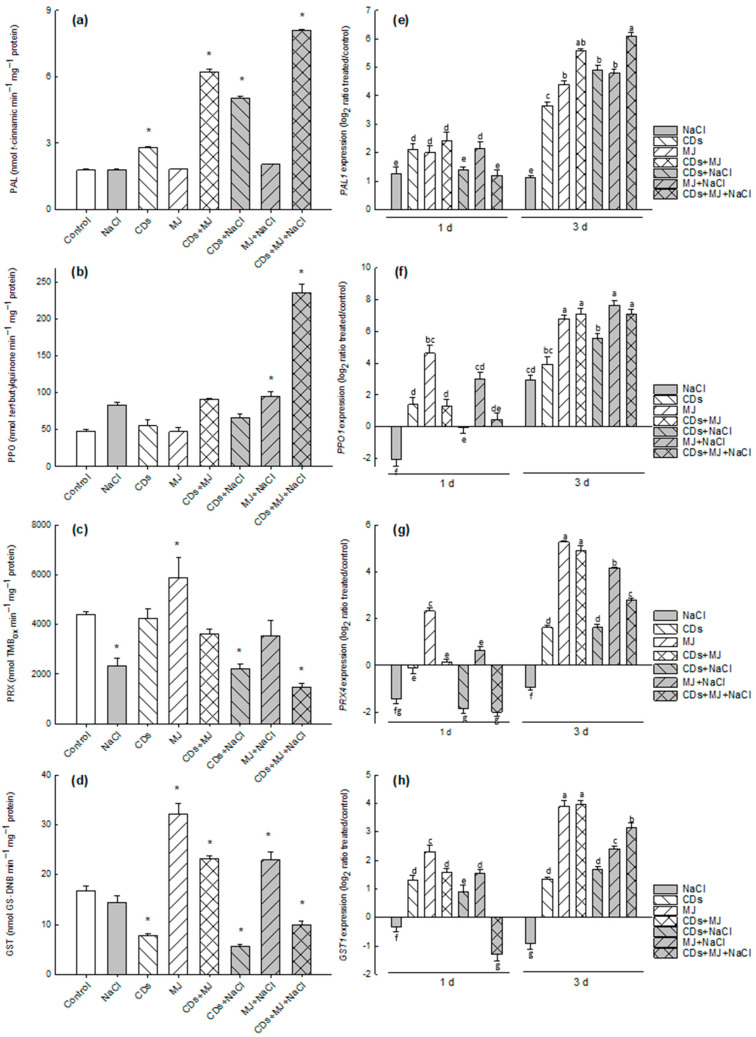
The effects of salt and/or elicitor treatments on the specific enzymatic activity of PAL (**a**), PPO (**b**), PRX (**c**), and GST (**d**), and the gene expression of *PAL1* (**e**), *PPO1* (**f**), *PRX4* (**g**), and *GST1* (**h**) analyzed by qRT-PCR. The data represent the mean ± SE of three independent replicates. Enzymatic values marked with an asterisk are significantly different from the control (*p* < 0.05) by Tukey’s HSD test. The gene expression values followed by different letters are significantly different at *p* < 0.05 by Tukey’s HSD test.

To determine in which way elicitor treatments affect the phenolic pattern in cells, the enzymatic activities and gene expression of phenylalanine ammonia-lyase (PAL), polyphenol oxidase (PPO), and class III plant peroxidases (PRX), as well as glutathione-*S*-transferase (GST), which seems to be involved in the secretion of *t*-R [58], were analyzed. Figure 3a,b show that the triple combination of CDs + MJ + NaCl resulted in a dramatic increase in both PAL and PPO activities (>4.5-fold compared to controls). In the rest of the treatments, both activities were in the range of the untreated controls, with the exception of PAL, in which 1.6-fold, 3.5-fold, and 2.8-fold increases were observed in CDs, CDs + MJ and CDs + NaCl treatments, respectively (Figure 3a), as well as in the case of PPO activity in the presence of MJ+ NaCl (2-fold compared with the control treatment) (Figure 3b).

PRX is actually the only plant enzyme that has been shown to catalyze the oxidation of *t*-R to viniferins [59]. Using *t*-R as substrate, PRX activity dropped in most NaCl treatments (ranging from 0.3- to 0.6-fold), increased in MJ-treated cells (1.6-fold), and remained closer to the control in CDs and CDs + MJ treatments (Figure 3c). Surprisingly, a similar PRX activity pattern was observed for all treatments using the non-physiological PRX-substrate tetramethylbenzidine (TMB) (Figure S1). GST activity was unaffected by NaCl alone treatments, decreased to half in the presence of CDs, and nearly doubled in MJ-elicited cultures (Figure 3d). The binary combinations of MJ, either with CDs or NaCl, increased GST activity, but to a lesser extent than MJ alone. The presence of NaCl in the medium tended to decrease GST activity in comparison to their respective non-saline treatments (Figure 3d).

**Figure 4:**
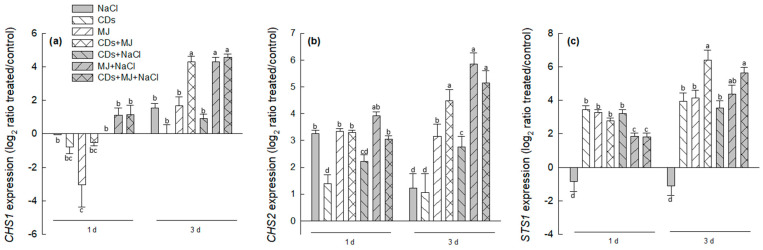
The effects of salt and/or elicitor treatments on the gene expression of *CHS1* (**a**), *CHS2* (**b**), and *STS1* (**c**) analyzed by qRT-PCR. The data represent the mean ± SE of three independent replicates. Values followed by different letters are significantly different at *p* < 0.05 by Tukey’s HSD test.

Based on previous studies [10,31,60] the relative expressions of specific genes (*PAL1*, *PPO1*, *PRX4*, *GST1*, *CHS1*, *CHS2*, and *STS1*) were analyzed by qRT-PCR after 1 day and 3 days of elicitation (Figure 3 and Figure 4). The expression of the *PAL1* gene was enhanced by the presence of elicitors after 1 day and showed a far greater increase after 3 days of treatment, particularly in CDs + MJ + NaCl-elicited cultures (>6-fold) (Figure 3e). The expressions of *PPO1* genes after 1 day of elicitation were up-regulated by NaCl treatments alone (>4-fold), but their induction was even more dramatic in the rest of treatments, particularly in MJ-treated cultures. Nevertheless, *PPO1* expression tended to decrease after 3 days of elicitation in all the treatments (Figure 3f). Conversely, the expression of the *PRX4* gene 1 day after elicitation was significantly reduced when cells were treated with NaCl alone or in combination with MJ or MJ + CDs. The *PRX4* transcript accumulation considerably increased in all treatments after 3 days, except for the treatment with NaCl alone. The maximal levels of *PRX4* expression were found in the presence of MJ, either alone or in combination with CDs or NaCl (~5-fold) (Figure 3g).

The expression of the *GST1* gene after 1 day of elicitation was enhanced by the addition of elicitors but decreased in NaCl and CDs + MJ + NaCl-elicited cultures (Figure 3h). After 3 days, a larger increase in *gst1* transcripts was found in all treatments, with the exception of salt-only treatments, in which *gst1* levels decreased.

The expressions of *CHS1* and *CHS2* genes, which control the first committed step for the flavonoid pathway, and *STS1*, which is involved in the biosynthesis of stilbene skeleton [10], were also analyzed. Figure 4a,b show that after 1 day of elicitation, *CHS1* expression was down-regulated in cells treated with NaCl alone or in the presence or MJ and/or CDs, whereas *chs2* transcripts remained relatively constant in all treatments (>3-fold), with the exception of CDs and CDs + NaCl treatments, in which the increase was less pronounced. Maximal levels of *CHS1* and *CHS2* expression were found in all MJ-elicited cultures after 3 days of elicitation. The transcript accumulation of the *STS1* gene displayed an expression profile similar to that observed for *PAL1*, with the exception that the *STS1* gene was down-regulated in NaCl alone treatments (Figure 4c).

### 3.4. Effects of Elicitors on ROS-Scavenging Enzymes under Salt Conditions

**Figure 5:**
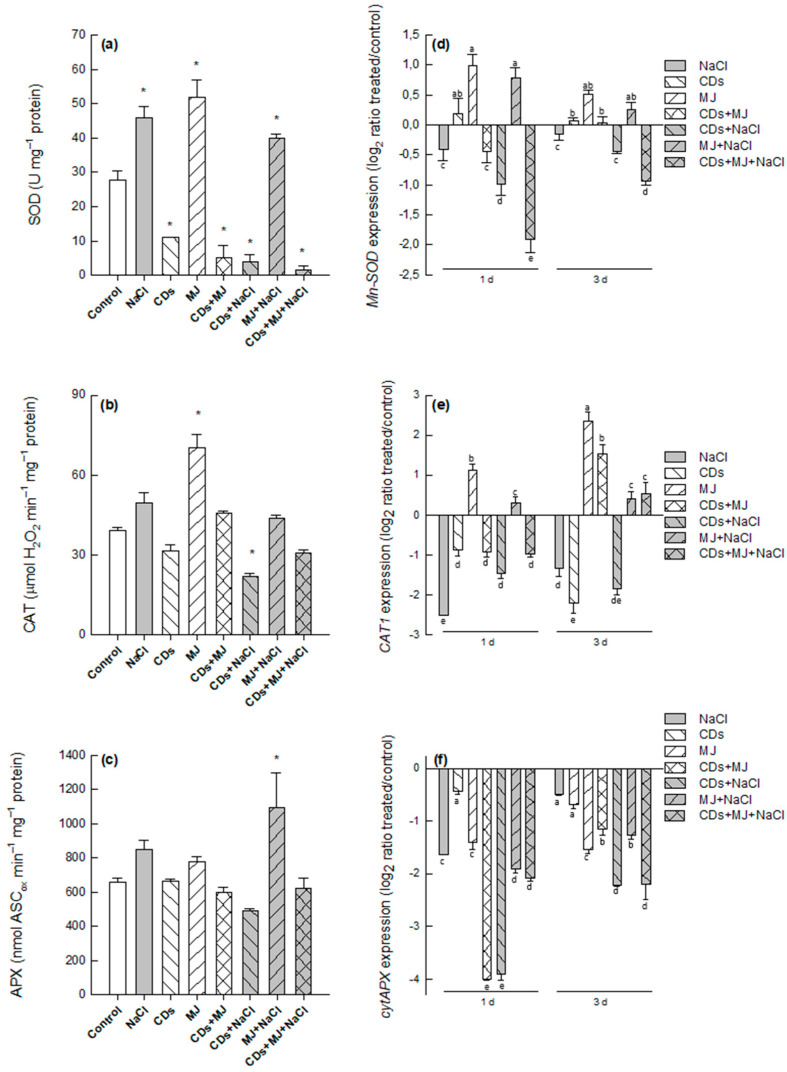
The effects of salt and/or elicitor treatments on the specific enzymatic activity of SOD (**a**), CAT (**b**), and APX (**c**) and the gene expression of *Mn-SOD* (**d**), *CAT1* (**e**), and cyt*APX* (**f**), analyzed by qRT-PCR. The data represent the mean ± SE of three independent replicates. Enzymatic values marked with an asterisk are significantly different from control (*p* < 0.05) by Tukey’s HSD test. Gene expression values followed by different letters are significantly different at *p* < 0.05 by Tukey’s HSD test.

To have more information about the ROS-scavenging capability of grapevine cell cultures, the changes induced by elicitors in the activities of SOD, CAT, and the ASC-GSH cycle were analyzed. Single NaCl treatments increased the activities of all the enzymes tested, although the differences only reached statistical significance for SOD (Figure 5a and Figure S1). The response pattern to elicitors was somewhat different, since SOD and CAT activities tended to increase under MJ treatment but to drop in CDs-elicited cultures. The presence of NaCl in the medium provoked a further decrease in the activity of SOD and CAT in elicited cultures, making this decrease more pronounced for SOD, particularly in the triple (CDs + MJ + NaCl) treatments (Figure 5a,b). None of the ASC-GSH cycle enzymes were significantly altered by salt or elicitors treatments, although their levels tended to increase in salt and MJ-treated cultures (Figure 5c and Figure S1). Additionally, a similar trend was observed for APX, which catalyzes the H_2_O_2_-dependent oxidation of ASC to monodehydroascorbate (MDHA), and MDHAR, which transforms MDHA into ASC using NAD(P)H as a reducer (Figure S1), whereas no DHAR activity was observed. It is interesting to note that the MDHAR activity found in grapevine SCC was high (~790 nmol NADH_ox_ min^−1^ mg^−1^ protein) in comparison with the values reported in the literature (200–500 nmol nmol NADH_ox_ min^−1^ mg^−1^ protein) [61]. This result suggests that MDHAR was effective enough to regenerate ASC before MDHA can disproportionate non-enzymatically to ASC and dehydroascorbate (DHA).

Several authors have reported that Mn-SOD, CAT1, and cytosolic APX isoforms are highly responsive to environmental cues, including salt stress [62,63,64]. The expression of these specific genes was analyzed by qRT-PCR after 1 and 3 days of elicitation. The analysis of mRNA expression revealed that *Mn-SOD*, *CAT1*, and *cyt-APX* genes were down-regulated upon NaCl treatments (Figure 5d–f). Only elicitation with MJ led to a slight increase in the expression of *Mn-SOD* and *CAT1* genes, an effect that was less pronounced in the MJ + NaCl treatments. Conversely, the accumulation of *Mn-SOD* and *CAT1* transcripts decreased in both CDs + MJ and CDs + MJ+ NaCl treatments. At day 3, a reduction in the expression of the three genes analyzed was observed. It is interesting to highlight that all the treatments appeared to down-regulate the expression of cytosolic APX (Figure 5f).

### 3.5. Principal Component Analysis of Cell Growth, ROS and Phenol Metabolism Parameters

**Figure 6:**
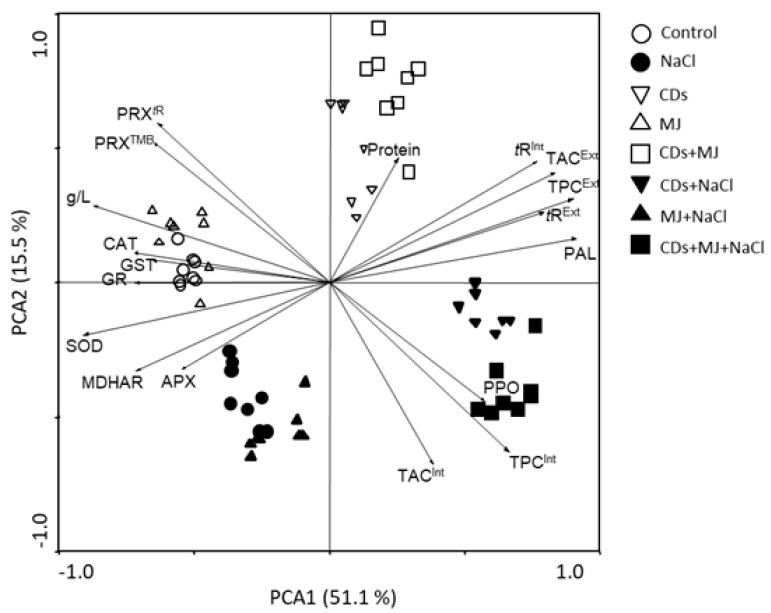
A principal component analysis (PCA) based on the correlation matrix applied to biochemical and enzymatic activities data sets. The non-saline and saline treatments are represented by unfilled and filled symbols, respectively. Each variable is represented by an arrow; the longer the length of the arrow, the greater its contribution to a given component. The angle between arrows indicates the degree of correlation among variables; the smaller the angle, the higher the correlation.

Finally, a PCA analysis was carried out to visualize the metabolic changes that occurred under the different treatments (Figure 6). The first component, which explained ~51% of the total variance, was mainly influenced on the positive side by *t*-R, TPC, and PAL activity, and by cell biomass (g L^−1^), piceid, and antioxidative enzymes on the negative side of the *X*-axis. PC1 clearly separates the untreated controls from CDs and CDs + MJ salt-treated cultures, as well as from MJ and salt single treatments. PCA2, which accounted for ~15% of the variation, was mainly associated with HCAs and (+)-catechin on the positive side and by protein contents on the negative side of the *Y*-axis. PCA2 separates MJ salt-treated cultures from CDs and CDs + MJ non-saline treatments. The five major clusters found in PCA suggest that the final response of Monastrell cell culture under both saline and non-saline conditions might be considerably modulated by the presence of CDs.

## 4. Discussion

It is well-known that ROS act in a similar way to a double-edged sword, as when they are present at low or moderate concentration, below the threshold level, they act as secondary messengers and participate in redox signaling pathways within the cells, inducing responses to overcome stress situations. The regulatory network comprising non-enzymatic and enzymatic antioxidant systems try to maintain and limit ROS levels within the cells, so that they are not harmful [65]. However, under stress conditions, ROS are generated in high concentrations, which can become toxic as they exceed the potential capacity of antioxidant systems and disturb cellular redox homeostasis [23]. The production of ROS in plant cells depends on the equilibrium between their production and detoxification systems [23]. However, the specific defense pathways that alleviate ROS-mediated stress have not yet been fully elucidated. Understanding the effect of salt stress on plant cell metabolism is of critical importance for deciphering the defense-related responses that can be used to improve plant growth and nutritional quality under stressful conditions. Here, we used a highly productive *t*-R grapevine SCC as a model system to analyze how salt treatment alters the expression/activity of key enzymes on both phenolic and antioxidative metabolism on CDs and/or MJ-elicited Monastrell SCC.

### 4.1. Salt-Treated Elicited-Cultures Exhibit Higher Intra- and Extra-Cellular Antioxidant Properties, Particularly in the Presence of CDs

Our results indicate that, at the dose applied, neither elicitors nor saline treatments induce cell death in Monastrell SCC, although a significant reduction of cell biomass accumulation was found after 4 days in all treatments (Figure 1). This reduction in cell biomass, which constitutes a weight loss of around 25%, could be due to a secretion of specialized metabolites towards the apoplast, which in the biological system used (SCC), is constituted by the extracellular medium in response to the presence of elicitors or even a slight loss of cellular water due to saline treatment. MJ as a signal molecule is known to be involved in the induction of the cellular defense systems, in the reprogramming of secondary metabolism, and in the arrest of the cell cycle [38]. Changes in cell division and expansion have also been previously observed in grapevine SCC exposed to NaCl [28], CDs, and/or MJ [10,66]. The reduction in cell growth is considered the result of a metabolic competition between growth-related and defense-related processes in response to stress conditions as well as elicitor/hormone treatments [67,68]. It is well known that plant survival under stress requires not only the control of cell growth patterns, but also the activation of the antioxidative network to ensure cellular redox homeostasis and to maintain cellular metabolic functions [23]. These processes have been considered characteristic responses of both plants and SCC to exogenously applied elicitors [17,18,67]. In plants, cellular redox homeostasis is maintained by a complex network of antioxidant enzymes and metabolites that remove and keep ROS at basal non-toxic levels [23,69]. In this study, the analysis of TAC, which is considered an integrated parameter to estimate the full spectrum of low-molecular antioxidants present in biological samples [70,71], revealed an increase in intracellular TAC values in all salt-treated elicited cultures, particularly in the presence of CDs. CDs-elicited cultures in combination either with MJ or NaCl also exhibited the highest extracellular TAC values (Figure 2b), with *t*-R being the main phenolic antioxidant found in the spent media (Figure 2d). Due to their molecular structure and shape, CDs can form inclusion complexes with a wide range of molecules, including *t*-R [72]. The complexation of *t*-R with CDs can not only improve its stability in the spent media, but also avoid its degradation, what can explain the higher extracellular TAC values found in comparison with intracellular TAC in CDs-elicited cultures in combination with either MJ or NaCl (Figure 2a,b).

### 4.2. In Addition to t-R, the Intracellular Accumulation of HCAs, Catechin, and Piceid Differentially Changes in Response to Saline and/or Elicitor Treatments

Binary combinations of CDs and MJ are known to synergistically enhance the synthesis and production of *t*-R in Monastrell SCC [10]. Here, we also observed a synergistic effect of CDs and salt on the extracellular accumulation of *t*-R, reaching levels higher than 60 mg g^−1^ DW (Figure 2d). However, when it comes to intracellular *t*-R levels, this synergistic effect was not observed in CDs + NaCl treatments (Figure 2c). These results suggest that the biosynthetic pathway leading to *t*-R could be differently affected by salt and/or CDs treatments. An HPLC-MS analysis revealed the intracellular accumulation of (+)-catechin and HCAs in single MJ and all the NaCl-combined treatments and the only presence of HCAs in salt alone treatments, whereas in single CDs-elicited cultures, only *p*-coumaric was detected (Table 1). In the triple treatments, the lowest content of chlorogenic acid as well as the highest content of *p*-coumaric acid and ferulic acid were detected. Ferulic acid is considered a potent antioxidant due to its ability to form resonance-stabilized phenoxy radicals [73]. The high levels of cellular ferulic acid found in salt-elicited cultures could explain, at least in part, the higher TAC values found in cells (r = 0.9, *p* < 0.01, Table S2). The strong correlation between both intra- and extracellular TAC and TPC (Figure 2a,e and Figure 2b,f, respectively) found in response to saline and/or elicitor treatments indicates that cellular redox homeostasis under saline conditions is largely maintained by increasing the production of phenolic compounds. A slight-to-moderate enhancement in the production of bioactive phenolic compounds by salt treatment has also been described in *Orthosiphon stamineus* [74] and *Cassia acutifolia* cell cultures [75]. Saline treatment has also been reported to increase the production of alkaloids in *Rauwolfia serpentine* and *Solanum khasianum* hairy root cultures [76], although glucosinolate production in broccoli SCC was unaffected by salt exposure [77]. Unfortunately, although salt treatment alone increased the production of HCAs and *t*-R, their levels were too low to be used for biotechnological production in Monastrell SCC.

In grapevine SCC, the main stilbenes produced are *t*-R and piceid [8]. With the exception of MJ, where piceid levels were similar to the control (~60 μg g^−1^ DW, Table 1), piceid concentration decreased in elicited cultures and treated with salt in comparison to untreated controls. Interestingly, the lowest piceid content was detected in most of the CDs-elicited cultures (~14–18 μg g^−1^ DW). These results indicate that the glucosylation of *t*-R can be directly or indirectly altered by saline and/or CD treatments. These results contrast with those reported in grapevine cv. Gamay SCC, where no changes in the piceid content were observed upon elicitation with CDs or CDs + MJ [58].

### 4.3. The Higher Levels of Phenolics in Salt-Treated Elicited-Cultures Fit with the Highest Expression/Activity of PAL

The observed accumulation of phenolics (Figure 2 and Table 1) are consistent with the induction of the phenylpropanoid pathway. As PAL is the key gateway enzyme of the phenylpropanoid pathway, its regulation by environmental factors, hormones, and elicitors is well reported in the literature [10,33,78]. Here, phenolic compounds *t*-R and TPC seem to be related to the sustainable expression of the *PAL1* gene and a high PAL activity, particularly in the triple treatments (Figure 3a,b after 3 d). The maximum production of *t*-R was obtained in CDs + MJ and triple combination treatments (Figure 2c,d), which fits with the highest expression of the *STS1* gene (Figure 5c, after 3 d), in agreement with our previous results [10]. However, a lack of correspondence between *t*-R levels and *STS1* gene expression in NaCl alone treatments was found. This discrepancy could be the result of post-transcriptional and/or post-translational regulatory mechanisms, as well as of the involvement of other STS isoforms involved in *t*-R biosynthesis. In this context, it is worth mentioning that up to 48 putative *STS* gene sequences have been identified, with at least 32 of them being potentially functional, so the involvement of different STS isoenzymes in NaCl-treated cultures cannot be ruled out [79]. A synergistic interaction between both elicitors (CDs and MJ), either alone or in combination with NaCl, on *t*-R yield was observed (Figure 2c,d), which appears to be caused by an enhancement in the transcription of *PAL1* (Figure 3e) and *STS1* (Figure 4c) genes as well as by a higher PAL activity level (Figure 3a). Similarly, a synergistic interaction between MJ and salt was noticed in regards to the intracellular levels of (+)-catechin (Table 1), which was correlated with the highest levels of *chs2* transcripts found in MJ + NaCl treatments (Figure 4b). Likewise, a synergistic effect was found in all elicited cultures in the presence of salt on the accumulation of ferulic acid. Altogether, the results suggest that the combination of salt and/or elicitors differently alter phenylpropanoid gene expression, leading to a substantial diversion in the metabolic flux of the biosynthetic pathways that generate stilbenes, flavonoids, and HCAs.

### 4.4. PPO and PRX Exhibit an Opposite Pattern in Response to Salt and Elicitors Treatments

PRX and PPO enzymes can play a crucial role in shaping the phenolic profile in plant cells [80,81]. In this study, the response pattern to salt-elicited treatments was opposite for both enzymes; whereas PRX activity tended to decrease, PPO increased, especially in the triple combination treatments (Figure 3b,c). Consistently, the transcript levels of *PPO1* appeared to be more expressed in salt-elicited cultures as well as in MJ-treated cells (Figure 3f). The induction of *PPO* gene expression by MJ and changes in the kinetic behavior of PPO (from latent to an active form) have also been previously reported [82]. It is tempting to suggest that the highest PPO activity found in the triple combination treatment could be the result of a PPO conformational change to a catalytically more active form (Figure 3b). It has been reported that polymeric quinones generated by PPO could exhibit higher ROS-scavenging activities than their respective monomers [83]. It is feasible to think that the increased biosynthesis of phenolics and the shift in phenol metabolism triggered in response to the salt + CDs + MJ treatments (Table 1 and Figure 3) are aimed to provide more antioxidant capacity (Figure 2e) that can help to maintain ROS homeostasis.

As previously mentioned, phenolics can be also be oxidized by PRXs at the expense of H_2_O_2_. Here, we found that in cell extracts, soluble PRX activity and *PRX4* expression were induced by MJ and down-regulated under saline conditions (Figure 3c,g). Increased cellular PRX activity by MJ exposure has also been described in *Panax ginseng* SCC [84]. The rise of PRX activity in MJ treated-cells could be related, at least in part, to the oxidation of *t*-R to viniferins. Viniferins possess higher antifungal properties than *t*-R, and their production has been reported to increase in Monastrell SCC upon biotic elicitation [85] as well as in grapevine cv. Italia SCC treated with MJ [86]. Nevertheless, it is important to consider that PRXs are encoded by a large number of genes, and the different PRX isoforms are involved in a wide range of physiological functions, such as defense against stress, lignification, ROS metabolism, auxin catabolism, and cell elongation [87,88,89,90]. Due to the highly polymorphic nature of PRXs, the increased PRX activity can also be related to the presence of PRX isoforms involved in the control of cell elongation and differentiation. As observed in our PCA analysis, the untreated control samples were defined by soluble PRX activities (Figure 6). Taken together, these observations suggested that PRX-mediated metabolic processes could be differentially affected by saline and elicitor treatments. Differential patterns in mRNA expression for lignin-forming PRX and cationic PRX have been described in tobacco SCC under salt stress [91], and the alteration of PRX isoenzyme pattern and cell growth in Monastrell cell cultures upon elicitation with salicylic acid have also been reported [48], which are in line with our results.

Soluble GSTs are a family of multifunctional enzymes involved in the cellular detoxification of xenobiotics and protection against oxidative stress [92]. GSTs possess diverse ligand-binding activities that have been associated with the transport of flavonoids [92] and *t*-R [58]. In our study, neither GSH-conjugating activity nor *GST1* transcript expression correlated with *STS1* expression or *t*-R accumulation, indicating that the *GST* gene analyzed, *GST1*, is unlikely to be involved in *t*-R transport. This result is not surprising, given the large number of GST isoforms, up to 107, found in grapevines [93]. Nevertheless, a gene expression induction of *GST1* was well-correlated with a higher GST activity in most treatments with MJ (Figure 3d,h). This coincides, in part, with the higher levels of ferulic acid and catechin found in the treatments only with MJ, and in all those combined with salt. The induction of GSTs by biotic and abiotic stresses has been widely reported, which is consistent with their role in oxidative stress protection [94,95]. The GST activity in our PCA analysis was strongly associated with antioxidant enzymes (Figure 6). It is therefore tempting to suggest the involvement of GSTs in the cellular antioxidative defense system in Monastrell SCC, although more studies are needed to corroborate these results.

### 4.5. Salt and MJ Treatments Boost Antioxidant Enzyme Activities Whereas CDs Treatments Decrease Them

The regulation of ROS homeostasis is critical to allow ROS-triggered signaling that, depending on their concentration, timing, and localization, results in the activation of appropriate defense responses [23]. In plant cells, it is well established that SODs, CATs, and APXs play a pivotal role in ROS metabolism [23]. SODs catalyze the dismutation of superoxide radicals to yield O_2_ and H_2_O_2_, which can be further reduced to H_2_O by CATs and APXs. In particular, the Mn-SOD isoenzyme, which is predominantly found in mitochondria, peroxisomal CAT1, and cytosolic APX isoforms, is considered to play a major role under different stress situations, including salinity [96]. Here, the analysis of the enzymatic activity and gene expression of SOD and CAT reveal a different ROS-scavenging enzymatic capability in MJ- and CDs-elicited cultures. Both SOD and CAT enzymatic activities, as well as the transcript levels of Mn-SOD and CAT1, appeared to be more expressed in MJ- than in CDs-treated cultures (Figure 5). The higher activities of SOD, CAT, APX, MDHAR, and GR in MJ in comparison to CDs cultures could be related to the higher intracellular H_2_O_2_ levels previously found in MJ-elicited cells after 1 day of treatment in comparison with the H_2_O_2_ levels found in CDs-treated cells [24]. Saline treatments alone provoked an enhancement of ROS-scavenging enzymatic activities, even though not at the transcriptional level. This lack of correlation between gene expression and the activity of antioxidant enzymes is not surprising, given that these enzymes are often subjected to translational modifications [97] and that they can show altered sensitivities to their substrates, as happens for CAT and APX towards H_2_O_2_ [65]. In fact, different reports have proved that CAT, APX, and SOD can be also regulated by nitric oxide-derived post-translational modifications such as S-nitrosation and tyrosine-nitration [97,98]. Our PCA analysis further showed that antioxidative enzymes differentiate saline and MJ samples from the other treatments. These findings are also supported by the results previously described [28], where a link between salt signaling and the JA pathway was reported in grapevine SCC by the induction of gadolinium-sensitive calcium influx channels and JAZ/TIFY transcripts. Although there are few reports regarding the effects of MJ and CDs on antioxidant network in SCC, it has been found that MJ treatments induced the activity of antioxidant enzymes in *Scrophularia kakudensis* SCC [99] and correlated with the improvement of salt tolerance in German chamomile plants [100].

## 5. Conclusions

Our results indicate that the strengthening of the antioxidative network in response to CDs, as well as in salt and MJ treatments, is carried out by the activation of both non-enzymatic and enzymatic antioxidant pathways, though their relevance differs among treatments. The former tends to favor the accumulation of antioxidant phenolics, whereas the latter tends to boost antioxidant enzyme activities. These differences can be related to the ability of CDs to favor not only the accumulation of antioxidant phenolic compounds through the formation of inclusion complexes, but also to enhance their biosynthetic pathways.

## Figures and Tables

**Table 1:** The effects of salt and/or elicitor treatments on the intracellular accumulation of different phenolic compounds in Monastrell SCC. The data represent the mean ± SE of three independent replicates. Different letters in the same column indicate significant differences according to Tukey’s HSD test (*p* ≤ 0.05). nd: non-detected.

Treatment	Phenolic Compounds (μg g^−1^ Dry Weight)				
	*p*-Coumaric Acid	Chlorogenic Acid	Ferulic Acid	(+)-Catechin	Piceid
Control	nd	nd	nd	nd	59.3 ± 1.6 ^a^
NaCl	0.84 ± 0.04 ^b^	0.79 ± 0.09 ^c^	2.4 ± 0.85 ^e^	nd	25.5 ± 1.8 ^d^
CDs	0.83 ± 0.12 ^b^	nd	nd	nd	17.8 ± 1.7 ^d^
MJ	0.32 ± 0.09 ^c^	0.89 ± 0.06 ^c^	8.7 ± 0.26 ^d^	0.13 ± 0.01 ^c^	64.4 ± 6.1 ^a^
CDs + MJ	0.03 ± 0.02 ^d^	nd	nd	nd	36.0 ± 4.9 ^c^
CDs + NaCl	0.02 ± 0.06 ^d^	1.4 ± 0.23 ^b^	21.2 ± 1.6 ^b^	0.04 ± 0.01 ^d^	14.6 ± 0.11 ^d^
MJ + NaCl	0.27 ± 0.00 ^c^	2.7 ± 0.03 ^a^	11.7 ± 0.63 ^c^	0.39 ± 0.01 ^a^	46.5 ± 4.3 ^b^
CDs + MJ + NaCl	1.35 ± 0.09 ^a^	0.14 ± 0.02 ^d^	30.8 ± 1.4 ^a^	0.16 ± 0.01 ^b^	14.4 ± 3.2 ^d^

## Data Availability

The data is contained within the article and Supplementary Material.

## Supplementary Materials

The following supporting information can be downloaded at: https://www.mdpi.com/article/10.3390/antiox11020388/s1, Table S1: The list of gene-specific primers designed using Oligoanalyzer 3.1 software for real time RT-PCR. Table S2: The correlations between cell growth, ROS, and phenol metabolism parameters in Monastrell suspension cell cultures. Figure S1: The effects of salt and/or elicitor treatments on the specific activity of class III peroxidase (PRX), monodehydroascorbate reductase (MDHAR), and glutathione reductase (GR). PRX activity was determined using the artificial PRX-substrate tetramethylbenzidine (TMB).
